# Stress Evolution of Amorphous Thermoplastic Plate during Forming Process

**DOI:** 10.3390/ma11040464

**Published:** 2018-03-21

**Authors:** Qi Wu, Tomotaka Ogasawara, Nobuhiro Yoshikawa, Hongzhou Zhai

**Affiliations:** 1 Institute of Industrial Science, the University of Tokyo, 4-6-1 Komaba, Meguro-ku, Tokyo 153-8505, Japan; wuqi@iis.u-tokyo.ac.jp (Q.W.); ogasa@telu.iis.u-tokyo.ac.jp (T.O.); 2 State Key Laboratory of Mechanics and Control of Mechanical Structures, Nanjing University of Aeronautics and Astronautics, Yudao Street 29, Nanjing 210016, China; zhaihongzhou@nuaa.edu.cn

**Keywords:** amorphous thermoplastics, residual stress, modeling, forming process

## Abstract

Amorphous thermoplastics, as a type of engineering plastic material, are used in various industrial sectors. In order to manufacture high-performance products, it is important to optimize their forming process to mitigate residual stresses. However, stress in a plate is difficult to measure, therefore, modeling provides a powerful way to investigate and understand the evolution of stress. In this study, the forming process of a polyetherimide (PEI) plate was modelled using finite element analysis, and then validated through a comparison with a warpage experiment. This study reveals that the whole forming process can be divided into three stages by the glass transition temperature *T_g_* of the PEI. The second stage, corresponding to the plate cooling from above *T_g_* to below *T_g_*, contributes a large portion of the residual stress in a short time. The final residual stress, the magnitude of which is affected by the cooling rate and plate thickness, shows a parabolic distribution through the thickness of the plate. These important conclusions are beneficial for improving the quality of an amorphous thermoplastic plate, while allowing highly efficient production.

## 1. Introduction

Thermoplastics have attracted increasing attention from both academia and industry owing to their unique advantages of highly efficient production and recyclability compared to thermosetting plastics. Thermoplastics can be classified as semi-crystalline and amorphous. Polyetherimide (PEI), as a typical amorphous plastic, has been used to manufacture high-performance products, such as carbon fiber reinforced thermoplastics based on a PEI matrix for the aerospace industry [1].

The molding process of amorphous thermoplastic is typically completed in a couple of minutes. Reducing the molding time saves costs and enhances productivity, but typically leads to poorer quality, including both visible distortion, such as warpage and spring-in, and invisible internal damage [2]. These defects could affect product performance, shorten their lifetime, and could even induce catastrophic failure of the material structure. The intrinsic reason for this is thermally-induced residual stress. Residual stress inside a plate is difficult to monitor in situ, thus modelling, especially finite element analysis (FEA), provides a powerful and feasible method for researchers to understand the stress evolution process and its mechanism.

Liu conducted a two-dimensional FEA of thermally-induced stress and warpage of an amorphous plastic, based on a viscoelastic phase transform model [3]. Gu et al. conducted a similar two-dimensional analysis [4]. Li et al. calculated the stress on the basis of the theory of shells [2]. Kamal et al. used a three-dimensional FEA simulation to predict internal stresses of both amorphous and semi-crystalline thermoplastics, but did not clarify how the stress evolves [5]. More studies, in terms of injection-molded products, were reviewed by Molales et al. [6]. Apart from the injection molding method, a number of plastic-based products are manufactured by other techniques. For example, compression molding and tape fiber placement have been used to manufacture continuous fiber-reinforced thermoplastics. In these processes, the flow induced stresses can be neglected, but thermal stresses play a dominant role [7]. Chapman and Trende published their modeling results of semi-crystalline thermoplastic composites [8,9], respectively, although the stress evolution processes in their results are not in agreement.

In this study, we establish a three-dimensional FEA program comprising thermal-viscoelastic models to accurately examine the stress evolution process of an amorphous plate during its cooling phase in a mold. First, the material properties of PEI are obtained from various experiments, and the related theories and models for both thermal analysis and mechanical analysis are introduced in Section 2. Based on the numerical models, the FEA software is programmed and validated by comparison with a warpage experiment in Section 3. The thermally-induced mechanical changes, including volume shrinkage and modulus change, are then presented in Section 4. The FEA is comprehensively evaluated to show the stress evolution of the amorphous thermoplastic plate during the cooling phase in Section 5, followed by an explanation of its intrinsic principles in Section 6. In Section 7, concluding remarks are presented.

## 2. Theory

### 2.1. Polyetherimide

We used E-type PEI sheets, manufactured by Mitsubishi Plastic, Inc. (1-1 Marunouchi 1-chome, Chiyoda-ku, Tokyo 100-8252, Japan), in the following experiments to obtain the material data. Differential scanning calorimetry and dynamic mechanical analysis were conducted to evaluate its glass transition temperature that is approximately 210 °C. Differential scanning calorimetry and dynamic mechanical analysis were also used to obtain its specific heat capacity and thermal-viscoelasticity, respectively. A xenon lamp flash test and pressure-volume-temperature test were conducted to obtain its thermal conductivity and specific volume, respectively.

### 2.2. Thermal Analysis

The forming process comprises both thermal and mechanical analyses. The thermal simulation should be conducted first to model the temperature distribution in the material cooling phase. Equation (1) is the governing equation describing the heat transfer process in an amorphous thermoplastic plate.

(1) $$\rho \left(T\right)Cp\left(T\right)\frac{\partial T}{\partial t}=\nabla \left[k\left(T\right)\nabla T\right]$$

Unlike in other studies in which material parameters were considered to be constants [5,8,9], the density, thermal conductivity, and heat capacity used in Equation (1) are all temperature-dependent to accurately reflect the physical change. Temperature-dependent thermal conductivity and heat capacity are described by Equations (2) and (3), respectively [10]:

(2) $$k\left(T\right)=\{\begin{array}{c} k\left(T_{g}\right){\left(T/T_{g}\right)}^{b_{1}}\;\mathrm{if}\;T\leq T_{g}\\ k\left(T_{g}\right)\left(b_{2}-b_{3}T/T_{g}\right)\;\mathrm{if}\;T>T_{g} \end{array}$$

(3) $$Cp\left(T\right)=\{\begin{array}{c} Cp_{s}\left(298\;K\right)\cdot \left(b_{4}+b_{5}T\right)\;\mathrm{if}\;T\leq T_{g}\\ Cp_{l}\left(298\;K\right)\cdot \left(b_{6}+b_{7}T\right)\;\mathrm{if}\;T>T_{g} \end{array}$$

### 2.3. Mechanical Analysis

After the thermal analysis, the mechanical analysis, including the thermally-induced shrinkage and stress relaxation caused by the viscoelastic behavior, should be performed. The volume shrinkage induces residual stress, while the viscoelasticity releases a portion of the formed stress. The empirical Tait equation, shown in Equation (4), is used to describe the specific volume, as a function of temperature and pressure [11,12]. The temperature-dependent density in Equation (1), and the coefficient of thermal expansion, can also be deduced from the specific volume:

(4) $$V\left(T,P\right)=V_{0}\left(T\right)\left[1-C\ln\left(1+\frac{P}{B\left(T\right)}\right)\right],$$

where *V*_0_ and *B* are calculated from Equations (5)–(7):

(5) $$V_{0}\left(T\right)=\left\{ \begin{array}{l} b_{8l}+b_{9l}\left(T-b_{12}\right)\;\;\mathrm{if}\;T>T_{t}\left(P\right)\\ b_{8s}+b_{9s}\left(T-b_{12}\right)\;\;\mathrm{if}\;T\leq T_{t}\left(P\right) \end{array}\right.$$

(6) $$B\left(T\right)=\left\{ \begin{array}{l} b_{10l}\exp\left(-b_{11l}\left(T-b_{12}\right)\right)\;\mathrm{if}\;T>T_{t}\left(P\right)\\ b_{10s}\exp\left(-b_{11s}\left(T-b_{12}\right)\right)\;\mathrm{if}\;T\leq T_{t}\left(P\right) \end{array}\right.$$

(7) $$T_{t}\left(P\right)=b_{12}+b_{13}P$$

Unlike metallic materials that exhibit dominant elastic properties, plastic materials have obvious viscoelastic properties that are also temperature-dependent. Since the strain established in the cooling phase of the forming process is small and the rate of strain is slow, the polymer’s viscoplasticity is ignored in this research [13,14,15]. Equation (8) is a Maxwell model in a Prony series mathematical format, and is a typical expression of the master curve covering the extended time duration at a reference temperature. By using a suitable temperature-dependent shift factor, the relaxation time in Equation (8) changes. As a result, the viscoelasticity of the PEI can be predicted at any temperature by substituting Equation (9) into Equation (8). The viscoelasticity effect and the shift factor of the PEI have been comprehensively researched in [16]:

(8) $$E\left(t\right)=E_{\infty }+\sum_{i=1}^{n}E_{i}\left(e^{-t/\tau_{i}}\right)$$

(9) $$E(t,T)=E\left(t/A_{T},T_{ref}\right)$$

**Figure 1:**
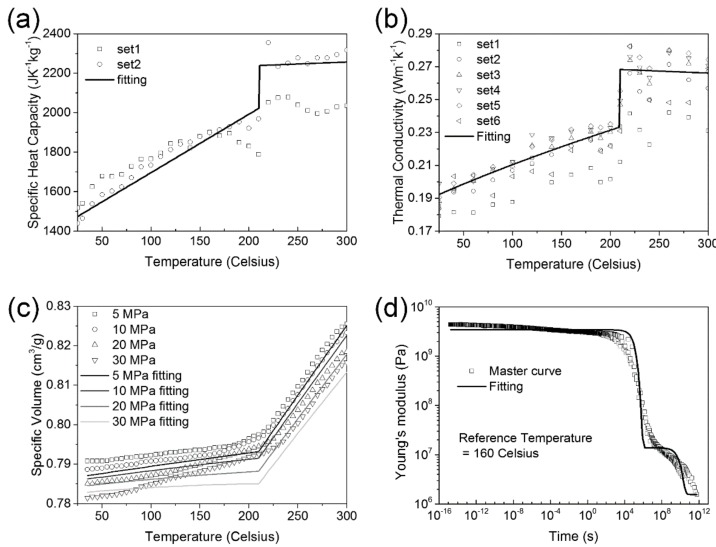
Material properties. (**a**) Specific heat capacity; (**b**) thermal conductivity; (**c**) specific volume; and (**d**) Young’s modulus.

The experimental data of specific heat capacity, thermal conductivity, specific volume, and Young’s modulus are plotted in Figure 1. The solid lines are the fitting curves of the corresponding material parameters. It is clear that all material properties show a knee point when the temperature is around *T_g_*.

## 3. Finite Element Analysis

### 3.1. Model

**Figure 2:**
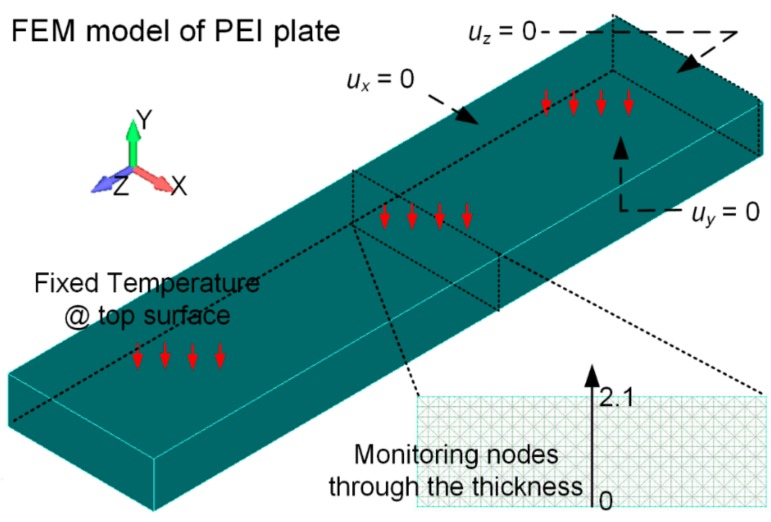
Finite element model of the PEI plate.

A 55.9 × 13.4 × 2.1 mm PEI plate was used in this study. Due to its symmetrical shape, only one quarter of the plate was modeled, and then meshed by tetrahedron elements, as shown in Figure 2. The number of nodes is 362,283, and number of elements is 252,518. In the thermal analysis, the temperature on the top surface of the plate was fixed to the ambient temperature for simplification. In the following mechanical analysis, the X- and Z-axes displacements on the left and back surface of the model were set to 0 to satisfy the symmetric boundary condition. Assuming the plate is compressed in the molding process, the mold will restrict its out-of-plane deformation until the mold is released after the temperature has cooled. Therefore, the Y-axis displacement on the bottom surface was also set to 0 in the cooling phase. By using these temperature and displacement boundary conditions, the following analysis can accurately reflect the forming mechanism of residual stresses in both the one-side cooling and the symmetric outside-to-inside cooling conditions. The monitoring points are the nodes through the plate thickness in the center of the model. The simulation was conducted on the self-made FEA platform, known as FrontCOMP_TP.

### 3.2. Program Validation

**Figure 3:**
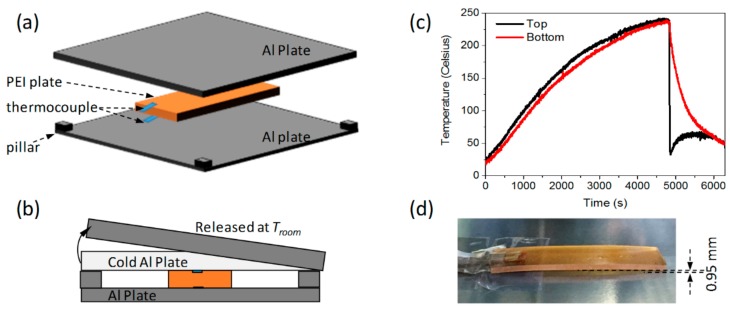
One-side cooling of PEI plate. (**a**) Exploded view of the schematic diagram of the experimental setup; (**b**) one-side cooling caused by cold aluminum plate; (**c**) temperature profiles on top and bottom surfaces; and (**d**) photograph of final warpage.

As stress is difficult to be measured in situ, we conducted a warpage experiment to validate our FEA platform. Figure 3a shows an exploded view of the schematic diagram of the experimental setup. On the top and bottom surfaces of the PEI plate, which is the same size as the FEA model, two thermocouples (Chino, C060-K) (32-8, Kumano-cho, Itabashi-ku, Tokyo 173-8632, Japan) were glued to record the temperature during the experiment. On the corners of a large aluminum plate, four small pillars, as tall as the thickness of the PEI plate, were installed. The PEI plate with this aluminum plate beneath it was heated to 235 °C. A further cold and heavy aluminum plate was placed on the top surface of the PEI plate to form a one-side cooling. As this aluminum-PEI-aluminum sandwich structure only restricted the warpage of the PEI plate, this experimental condition is analogous to the boundary conditions used in the simulation, as shown in Figure 3b. After the whole setup reached room temperature, the top aluminum plate was removed. Consequently, the formed residual stress distorted the plate and a warpage was observed. Figure 3c shows the smoothed temperature profiles on the top and bottom surfaces of the PEI plate. The warpage of the PEI plate, measured by a caliper, was 0.95 mm, as shown in Figure 3d.

**Figure 4:**
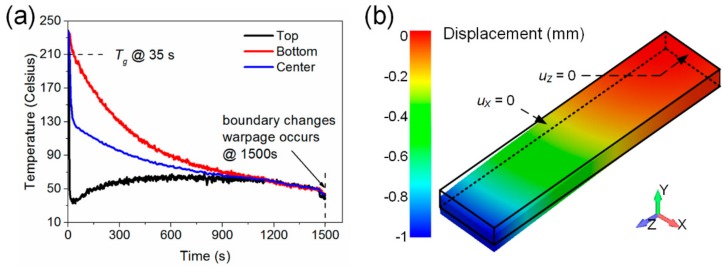
Simulation of one-side cooling of PEI plate. (**a**) Temperature profiles at different positions through thickness; and (**b**) simulated final warpage.

The FEA model defined in Section 3.1 was used for the simulation. The recorded temperature profiles in the cooling phase were used as the Dirichlet boundary in the thermal analysis. Figure 4a shows the temperatures on the top, center, and bottom of the plate. After the thermal analysis, the stress analysis was conducted by using the boundary conditions defined in Section 3.1. The fixed bottom surface is then set to release at 1500 s in order to simulate the released boundary constraint from the aluminum plate. The modeled warpage of the plate had a maximum displacement of 0.999 mm, which is close to the measured value. This experiment validated the program with the encoded physical models solidly.

## 4. Thermally-Induced Mechanical Change

### 4.1. Temperature Profile

**Figure 5:**
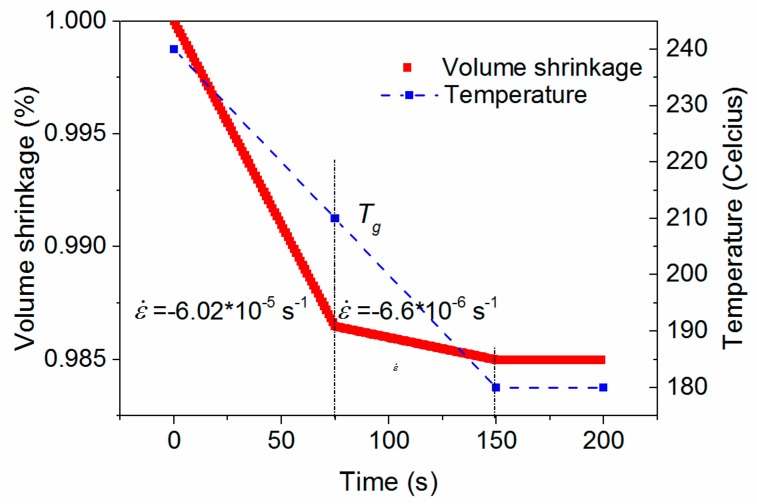
Temperature profile and corresponding volume shrinkage in the slow cooling condition.

In order to reveal the physical principles of the residual stress, a simplified ideal thermoplastic forming process is considered. In a typical manufacturing process, the PEI-based materials cool down in minutes from a temperature above *T_g_* to room temperature *T_room_*, typically 400–20 °C. In this model the maximum temperature and room temperature were set as 240 °C and 180 °C, respectively. Although this temperature range is small, it reflects the actual physical processes as it covers all the material phases, and exhibits all material changes. The temperature decreases linearly from 240 to 180 °C in 150 s, and then remains constant up to 200 s, as shown in Figure 5.

### 4.2. Volume Shrinkage

The red line in Figure 5 is the corresponding volume calculated from Equations (4)–(7) when the pressure is set to 0.1 MPa, as any pressure effect is outside the scope of this study. For an isotropic material, like PEI, its one-directional thermally-induced strain is one third of the volume shrinkage. Therefore, the corresponding rate of strain can be calculated from Equation (10), and is shown in Figure 5. The volume shrinkage curve has three sections. Before 75 s, when the temperature is higher than *T_g_*, the rate of strain is high and approximately linear with a value of −6 × 10^−5^ s^−1^. From 75 to 150 s, the rate of strain decreases to −6.6 × 10^−6^ s^−1^ albeit with linear slope when the temperature is lower than *T_g_*. After 150 s, no more shrinkage occurs since the temperature keeps constant:

(10) $$\dot{\varepsilon }=\frac{dV}{3Vdt}$$

### 4.3. Modulus and Shift Factor

**Figure 6:**
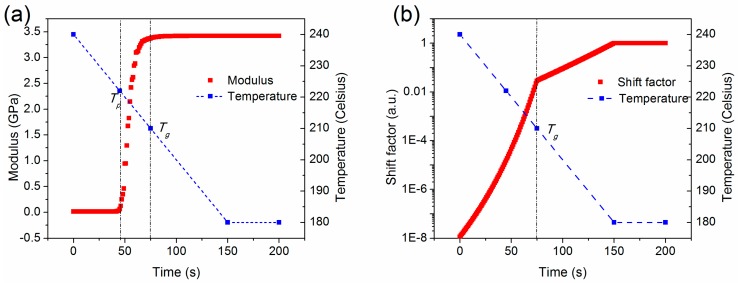
Mechanical property changes during cooling process. (**a**) Young’s modulus; and (**b**) shift factor.

Figure 6 shows the change of the PEI mechanical properties in the cooling phase. The PEI Young’s modulus is largely temperature-dependent, as shown in Figure 6a. When the temperature is higher than the rubbery plateau temperature *T_p_*, its Young’s modulus is small as the PEI is in its rubbery region. When the temperature is lower than *T_p_*, but higher than *T_g_* (in the range of approximately 220 °C–210 °C), the PEI enters the leathery region, and its Young’s modulus increases significantly. After 75 s, when the temperature is less than *T_g_*, its Young’s modulus remains approximately constant at a large value of 3.4 GPa. Moreover, the PEI stress relaxation time is also influenced by the temperature, and its temperature-dependent shift factor is shown in Figure 6b at a reference temperature of 180 °C. The shift factor increases exponentially when the temperature decreases linearly to *T_g_*. Below *T_g_*, the rate of increase of the shift factor decreases, and the value of shift factor approaches 1. As a result, the stress relaxes in an extremely short time at high temperatures, e.g., greater than *T_g_*. In contrast, the PEI at low temperatures is analogous to a purely elastic material. Both Young’s modulus and the shift factor have significant impacts on the stress evolution.

## 5. Stress Evolution

### 5.1. Residual Stress Distribution

The volume shrinkage, Young’s modulus, and shift factor curves shown in Figure 5 and Figure 6 are only applicable to items with uniform temperature distributions. The only condition that satisfies the above is to assume that the PEI sheet is sufficiently thin, and the temperature change is sufficiently slow. In an actual component, this becomes complex due to uneven temperature distributions through its thickness.

**Figure 7:**
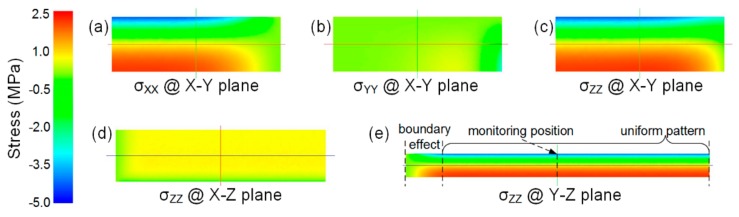
Contour of stress distribution on different planes. (**a**–**c**) σ_xx_, σ_yy_, and σ_zz_ on the X-Y plane; (**d**,**e**) σ_zz_ on the X-Z and Y-Z planes.

By using the FEA model, and the temperature and displacement boundary conditions introduced in Section 3.1, together with the input temperature profile in Figure 5, the thermal stresses in the modeled PEI plate are obtained. Figure 7a–c shows the stresses distributed in the X–Y plane after 200 s, the end of the cooling phase. The in-plane stresses σ_xx_ and σ_zz_ exhibit similar distributions. However, the out-of-plane stress σ_yy_ differs, with a smaller value. As the in-plane stresses are dominant, we focus on σ_zz_ in the following discussion.

The distribution of σ_zz_ on the X–Y, X–Z, and Y–Z planes in Figure 7c–e clearly show the boundary effect. For example, in Figure 7e, σ_zz_ exhibits a different pattern near the free boundary, but a uniform pattern in the areas away from the free boundary, including the monitoring position. Typically, the length and width of a plate-like plastic product are much greater than its thickness, so the monitoring position showing the uniform pattern of σ_zz_ is suitable to be used in the following analyses in order to avoid the boundary effect.

### 5.2. Temperature Distribution Through Plate Thickness

**Figure 8:**
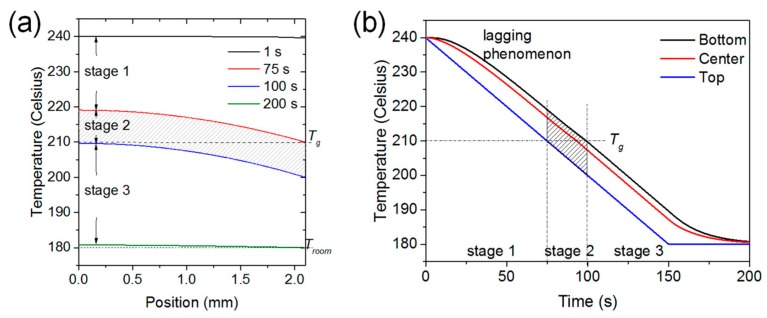
Temperature distribution through plate thickness at different times. (**a**) Temperature-versus-position curves at five different times; and (**b**) temperature-versus-time curves at three different positions.

Four typical temperature-versus-position curves at 1, 75, 100, and 200 s are shown in Figure 8a, where 0 and 2.1 mm on the X-axis are the bottom and top surfaces, respectively. The top surface of the PEI plate cools and reaches *T_g_* at 75 s. The plate then cools further until the bottom surface reaches *T_g_* at 100 s. The temperature of the entire plate decreases further, and approaches *T_room_*. These four curves divide the whole process into three stages, which can also be clearly identified in the temperature-versus-time curves in Figure 8b. In the actual manufacturing process, the duration of stages 1 and 3 will be significantly longer due to the larger temperature ranges used. The duration of stage 2 is affected by both the cooling rate and plate thickness, as it represents the time required by a plate to completely cool down from above *T_g_* to below *T_g_*, indicated by the gray-shadowed area in Figure 8.

### 5.3. Thermal Strain and Rate of Strain

**Figure 9:**
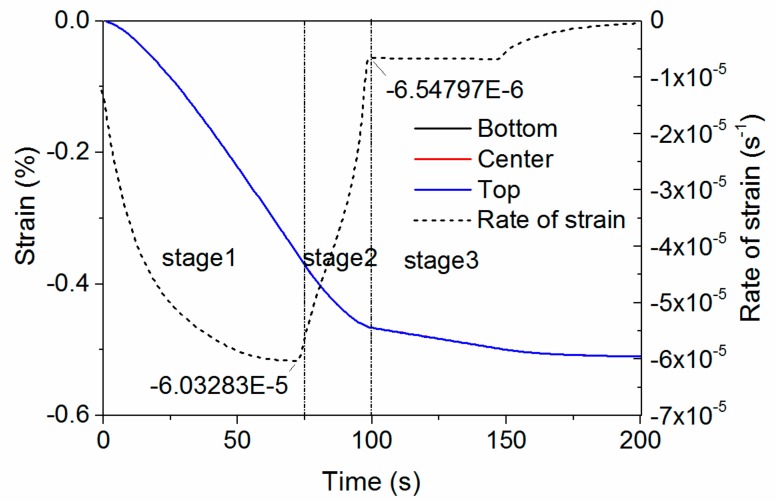
Thermally-induced strain and corresponding rate of strain.

The thermal strain on the bottom, center, and top positions are plotted in Figure 9. These three curves overlap, demonstrating that the plate has the same shrinkage through the thickness, although their temperature-versus-time profiles are different. This is contrary to straightforward thought that different temperature profiles will lead to different volume shrinkages. Moreover, these curves are not linear, differing from the volume shrinkage curve shown in Figure 5. The rate of strain is then calculated by Equation (11), and shown as the dashed line in Figure 9. The rate of the strain curve clearly exhibits three sections, with the same characteristic times as in Figure 8. The final rate of strain in stage 1, and the initial rate of strain in stage 3, are identical to the rate of strain shown in Figure 5 when temperature of the PEI was higher and lower than *T_g_*, respectively. The rate of strain gradually changes in stage 2 from −6.03 × 10^−5^ to −6.55 × 10^−6^ s^−1^.

(11) $$\dot{\varepsilon }=\frac{d\varepsilon }{dt}$$

### 5.4. Stress in Stage 1

**Figure 10:**
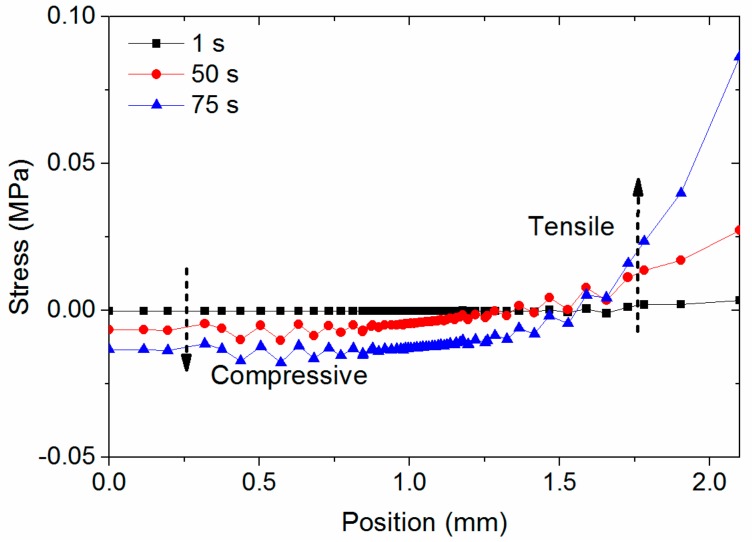
Stress evolution in stage 1.

Figure 10 shows the evolution of σ_zz_ in stage 1. The top surface of the plate shows tensile stress, while the bottom surface shows compressive stress. As the PEI closest to the top surface cools down first, ideally, that part will typically shrink before other parts. However, because of the identical displacement, the PEI inside, which is hotter and, thus, has a greater volume, prevents the outside cooler part from shrinking at the ideal rate of strain. Consequently, the nodes on the top surface show tensile stress and the nodes on the bottom surface show compressive stress. However, the values are negligible due to the low stiffness and short relaxation time of the material. When the PEI is in the rubbery region, its Young’s modulus is approximately two thousand times smaller than that in the glassy region. Thus, the formed stress will be negligible, as stress equals strain times Young’s modulus. In addition, even if a certain level of stress is formed, it will relax in a very short time when the temperature exceeds *T_g_*.

### 5.5. Stress in Stage 2

**Figure 11:**
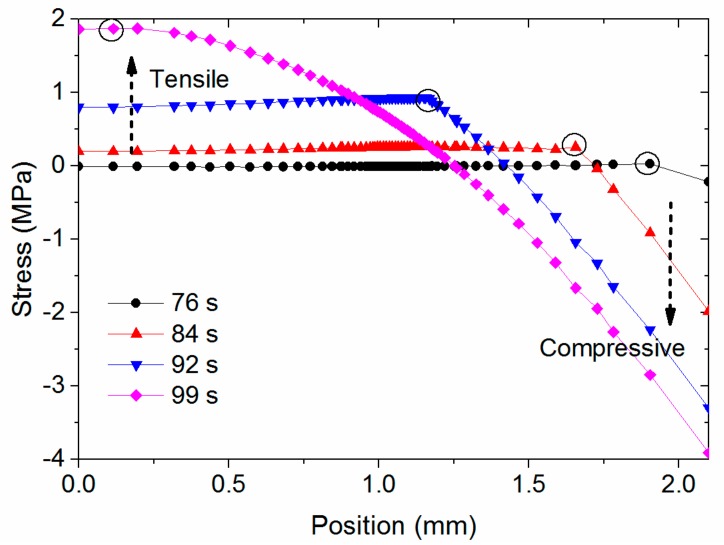
Stress evolution in stage 2.

**Figure 12:**
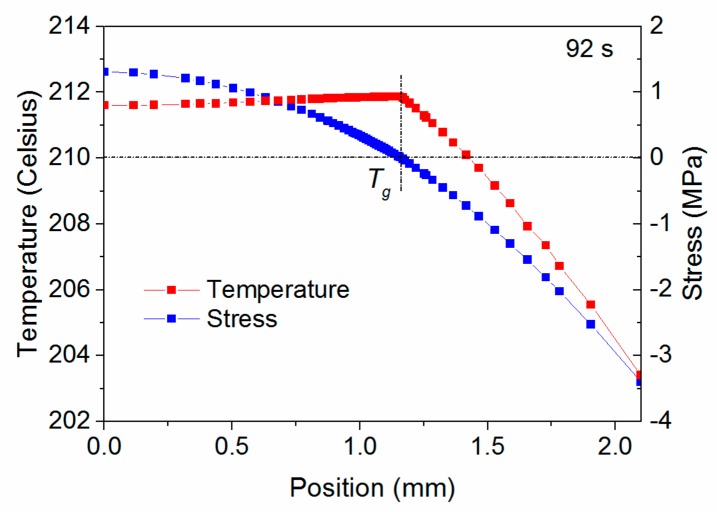
Knee point in curve corresponding to *T_g_*.

Figure 11 shows the complex evolution of σ_zz_ in stage 2. In general, the top surface shows compressive stress while the bottom surface shows tensile stress. In addition, there is a knee point showing the maximum tensile stress on each curve, indicated by the black circles. Figure 12 correlates the stress and temperature distribution at 92 s, a typical time in stage 2, and clearly shows that the knee point in the stress curve corresponds to the position of temperature *T_g_*. This knee point divides the stress curve into two parts. The relatively hot material has a temperature greater than *T_g_*, and is close to the bottom surface. The relatively cold material has a temperature lower than *T_g_*, and is close to the top surface. The relative hot PEI that is in the leathery region ideally shrinks more than that in the glassy region, as the ideal rate of strain of the PEI decreases when the temperature is less than *T_g_*. However, the relatively cold PEI obstructs the fast shrinkage of the hot PEI, i.e., the hot PEI promotes the shrinkage of the cold PEI, because of the identical thermal strain. As a result, the PEI exhibits an uneven stress distribution through the thickness. The value of the stress is determined by the rapidly increasing Young’s modulus when the PEI is in its leathery region. Furthermore, as the PEI in the glassy region has a lengthy stress relaxation time, the stress remains, and then accumulates to form a parabolic shape.

### 5.6. Stress in Stage 3

**Figure 13:**
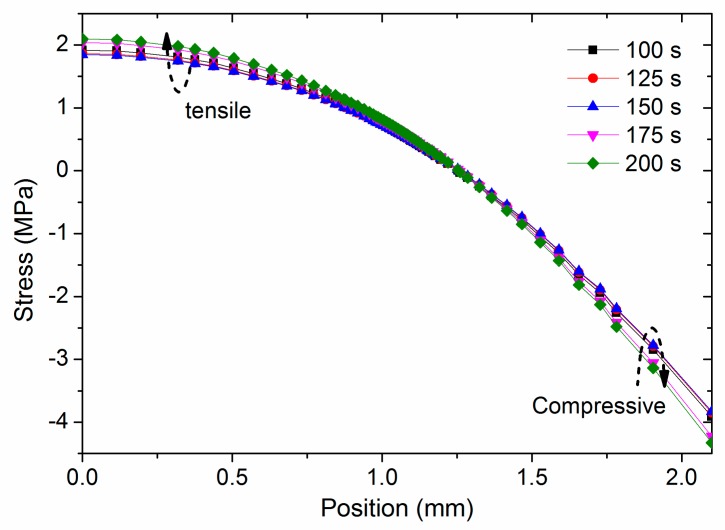
Stress evolution in stage 3.

Figure 13 shows the evolution of σ_zz_ in stage 3. From 100 to 150 s, there is the same rate of strain through the entire plate as the temperature is lower than the *T_g_* throughout, and the bottom and top surfaces cool at the same rate. Thus, neighboring areas do not restrict interaction. Both the tensile and compressive stresses then decrease due to the stress relaxation. After 150 s, the PEI near the top surface no longer shrinks, as its temperature remains constant at *T_room_*. However, the PEI near the bottom surface continues to shrink marginally more. Therefore, the tensile and compressive stresses increase slightly. However, as the PEI is in the glassy region with high modulus and long relaxation times, and also because the temperature difference between the top and bottom surfaces are small in this simulation case, the residual stress change is insignificant in stage 3.

### 5.7. Stress Evolution Process

**Figure 14:**
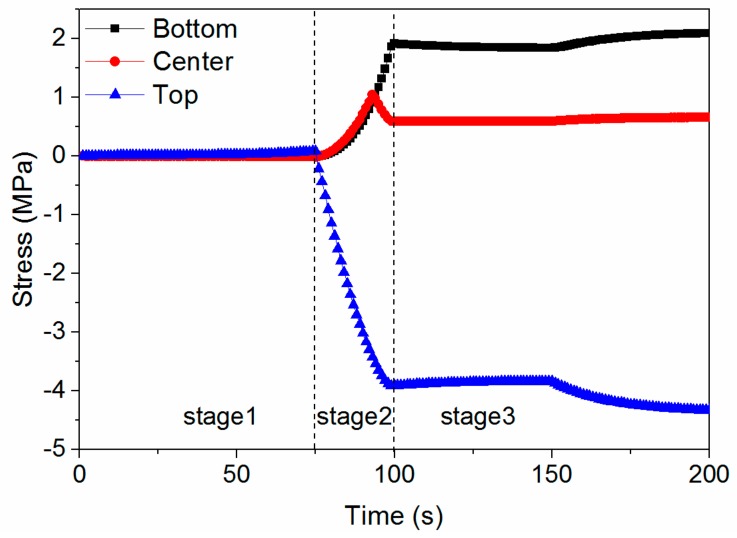
Stress evolution at different positions.

Figure 14 shows the stress evolution at different positions. The final residual stresses at the top and bottom surfaces are −4.33 and 2.09 MPa, respectively. The stress in stage 1 remains approximately constant. Therefore, stage 1 does not have any impact on the residual stress although it is a lengthy stage, considering that the process of practical manufacturing begins from 400 °C. The stress formed in stage 2 is more apparent than in the other stages. Therefore, although stage 2 is of short duration, reducing the stress formed in stage 2 can effectively mitigate the residual stress of the final product. In stage 3, the stress mitigation caused by stress relaxation is negligible, as the major stress change comes from the temperature difference between the top and bottom surfaces. Thus, in an actual case, the stress formed in stage 3 may be greater than the simulated stress in the simplified condition.

## 6. Forming Mechanism of Residuals Stress

**Figure 15:**
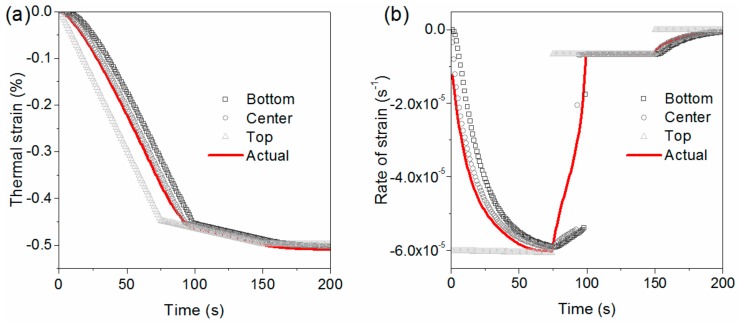
Difference between ideal shrinkage and actual shrinkage. (**a**) Ideal thermal strain at different positions and actual strain; and (**b**) the ideal rate of strain and actual rate of strain.

When a plate in a mold is cooled, the temperature through its thickness is not uniform, and is influenced by the cooling rate and the plate thickness. Amorphous thermoplastic, with different temperatures through the thickness, is ideal to have different thermal strains at different positions. This could lead to different rates of strain, such as a PEI in stage 2. However, the thermoplastic will have identical shrinkage, as it is constrained in the mold. The actual shrinkage is also determined by the temperature distribution and the material properties. Figure 15 shows the difference between the ideal and actual cases clearly. The shaded gray dotted lines in Figure 15a are from the ideal thermal strains $\varepsilon_{ideal}$ calculated by directly substituting temperature curves in Figure 8b into Equations (4)–(7). The red line is the actual thermal strain $\varepsilon_{actual}$, and differs from the ideal lines.

The time-related strain and stress are connected by a constitutive equation for linear viscoelasticity, as shown in Equation (12) [16]:

(12) $$\sigma \left(t\right)=\int_{-\infty }^{t}E\left(t-t'\right)\dot{\varepsilon }\left(t'\right)dt'$$

This well-known equation is based on the principle that the effects of sequential changes in strain are additive, i.e., it is a function of rate of strain. However, the ideal thermal strain derived from plastic shrinkage is a spontaneous motion, and does not induce any stress. Only when plastic at a certain position, interacting with neighboring plastic, is compulsively stretched or compressed, can stress be formed. Thus, the strain in Equation (12) is the difference between $\varepsilon_{ideal}$ and $\varepsilon_{actual}$, as expressed in Equation (13):

(13) $$\varepsilon =\varepsilon_{ideal}-\varepsilon_{actual}$$

By substituting Equations (11) and (13) into Equation (12), Equation (14) can be obtained:

(14) $$\sigma \left(t\right)=\int_{-\infty }^{t}E\left(t-t'\right)\left({\dot{\varepsilon }}_{ideal}\left(t'\right)-{\dot{\varepsilon }}_{actual}\left(t'\right)\right)\;dt'$$

**Figure 16:**
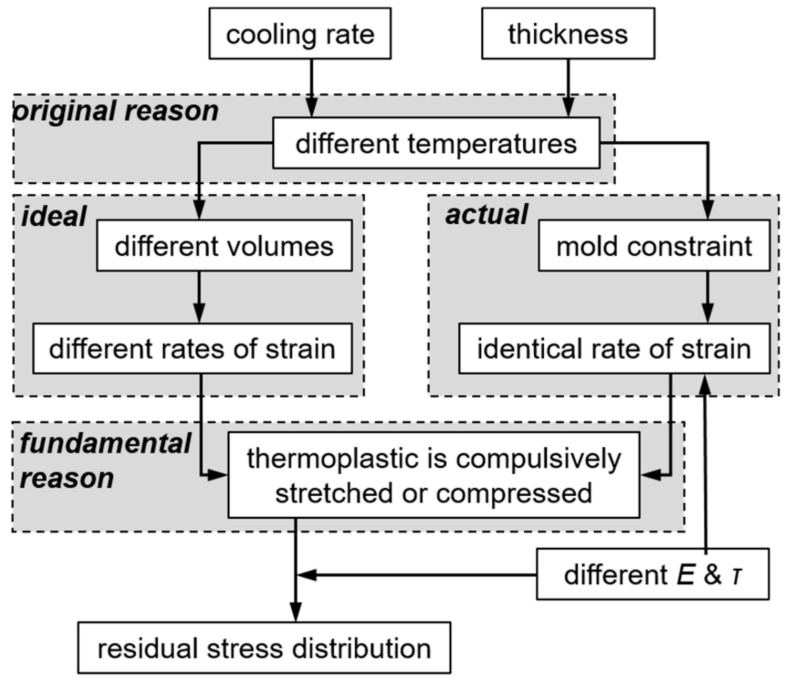
Forming mechanism of residual stress.

Equation (14) depicts that stress is related to the conflict between the ideal and actual rate of strain, as well as related to the duration and time-dependent Young’s modulus. The gray lines in Figure 15b are the rates of strain at different positions corresponding to the thermal strain shown in Figure 15a. Therefore, there is an obvious difference between the ideal and actual rate of strain, especially in stage 2. Thus, a large part of residual stress forms and accumulates in stage 2, to finally form a skin-to-core distribution. Figure 16 shows the explanation of the mechanism of the residual stress evolution when the amorphous plastic is in a mold.

From the above analysis, we conclude that the original cause of residual stress is due to temperature differences in a plate. However, the fundamental reason are the different rates of strain. The rates of strain differences are derived from the temperature differences, but the temperature differences did not all induce the differences in the rate of strain. For example, although there is a 10 °C temperature difference from 100 to 150 s in Figure 8, there is no obvious difference in the rate of strain shown in Figure 15. Consequently, there is no stress growth in this period.

Furthermore, Equation (14) indicates that both the cooling rate and the plate thickness will affect the final stress. As the stress formed in stage 2 is the dominant stress in the simulation, we use this stage as an example. At similar durations of stage 2, a higher cooling rate, corresponding to a higher rate of thermal strain, will result in greater residual stresses. At similar cooling rates, a shorter duration that will be influenced by the plate thickness will result in less accumulation of residual stress. Roughly, duration times the cooling rate equals the temperature difference. Therefore, the greater the temperature difference between the top and bottom surfaces of the thermoplastic plate, the greater the residual stress will be. In the appendix, we change the cooling rate and plate thickness to verify this.

## 7. Conclusions

The stress evolution of an amorphous thermoplastic plate in a one-side cooling condition is modeled in this study. The cooling phase can be divided into three stages.

Stage 1: When the temperature of the whole plate is greater than *T_g_*, stress barely forms.
Stage 2: When the plate cools to *T_g_* from one side to the other side, a large portion of the residual stress forms in a relatively short time, although the duration of this stage is typically short.
Stage 3: Until the whole plate cools to room temperature, the residual stress changes further, and finally a parabolic-shaped residual stress forms.

We evaluated the physical process, and found that the different rates of strain induced by different temperatures are the dominant and fundamental factors determining the residual stress. The findings of this study enhance the knowledge of residual stress evolution, and also has the potential to optimize the forming process.

## Figures and Tables

**Figure A1:**
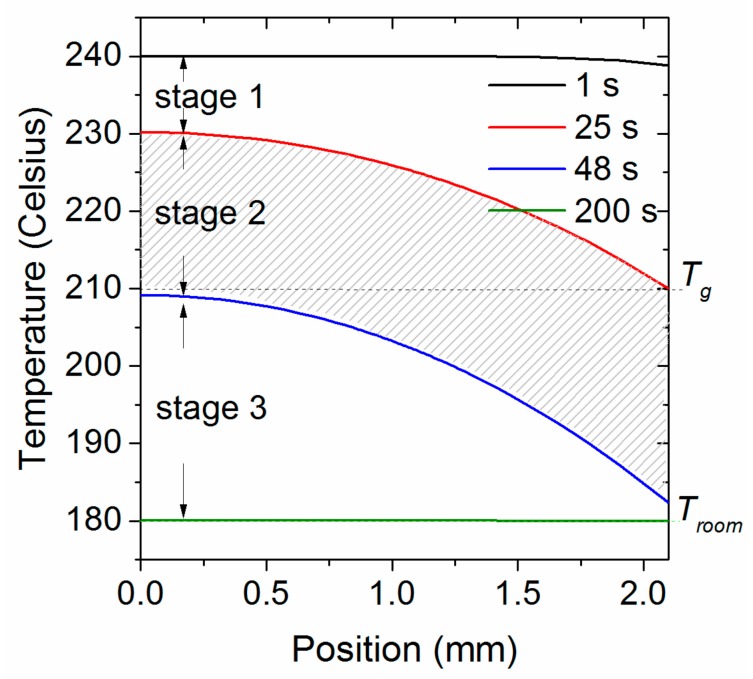
Temperature-versus-position curves of fast cooling rate case at four different times also divided the whole process into three stages.

**Figure A2:**
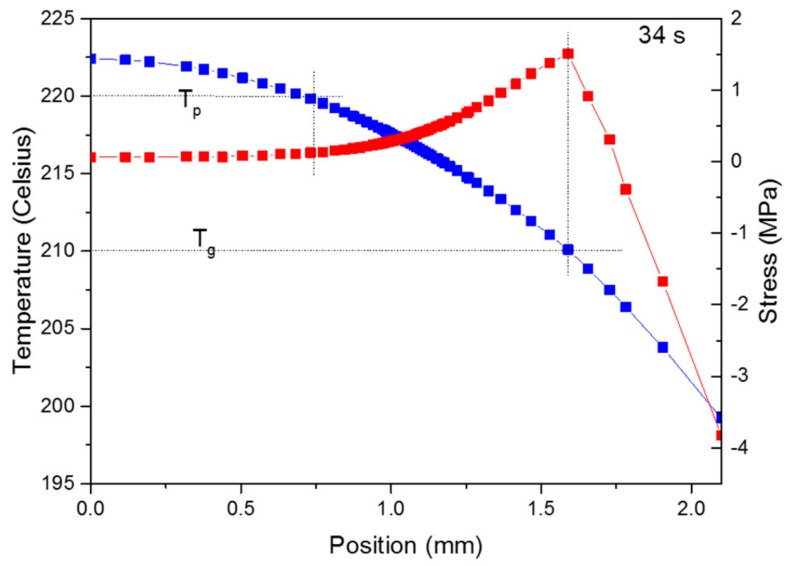
Knee point in stress curve corresponds to *T_g_*, and point where stress begins to develop corresponds to *T_p_*.

**Figure A3:**
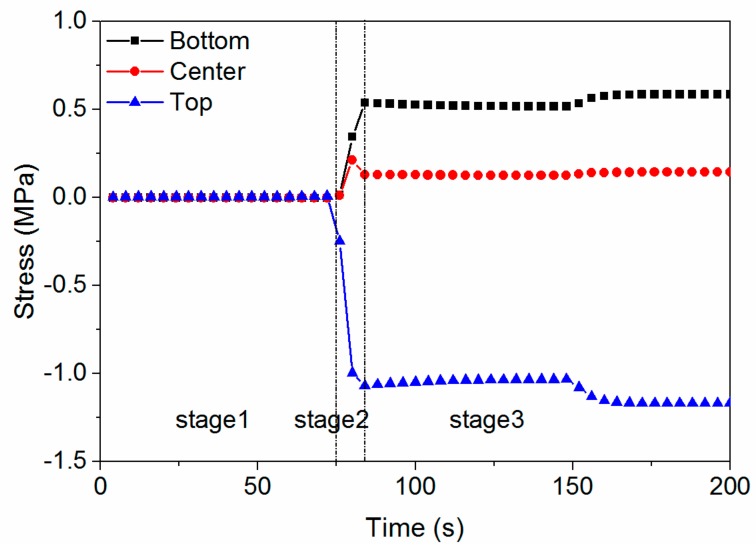
Stress evolution curve of the thin plate.

## Nomenclature

*T*	Temperature, K
*T_g_*	Glass transition temperature, 483 K
*T_room_*	Room temperature, 298 K
*T_t_*	Pressure-dependent T_g_
*T*	Time, s
*V*	Volume, m^3^
*V_0_*	Reference volume, m^3^
*P*	Pressure, MPa
*ρ*	Density, kg/m^3^
*Cp*	Specific heat, kJ/(kg·K)
*k*	Thermal conductivity, W/(m·K)
*B*	Material coefficients
*C*	Constant in Tait equation, 0.0894
*A_T_*	Shift factor
*E*	Young’s modulus, MPa
*τ*	Stress relaxation time, s
*ε*	Strain
*σ*	Stress, MPa
*b*	Fitting parameter

Subscript *i* represents the term, *l* and *s* represent the liquid and solid states, respectively.

## Appendix A. Cooling Rate

A case with a fast cooling rate, while keeping the same thickness of the PEI plate, is considered. The Dirichlet temperature boundary decreases from 240 to 180 °C in 50 s, three times faster than the cooling rate in Section 5. Figure A1 shows the four temperature-versus-position curves, which divide the process into three stages. As opposed to the slow cooling case where the top and bottom surfaces had an approximately 10 °C difference in stage 2, the fast cooling results in an approximately 20 °C temperature difference.

In the stress analysis, different positions have identical thermally-induced strains, although their temperatures are different. This phenomenon is similar to that shown in Figure 9. The evolution of σ_zz_ also exhibits three stages. Stages 1 and 3 are analogous to those of the slow cooling case. The stress evolution in stage 2 also contributed to the major portion of the residual stress, but the stress-versus-thickness curve differs slightly. Figure A2 shows the correlation of stress and temperature through the thickness of the plate at 34 s, a typical time in stage 2. The knee point in the stress curve is a peak, which means the stress increases from the bottom surface to the peak point. This differs from Figure 11 and Figure 12, where the stresses at positions between the knee point and bottom surface have approximately similar values. It can be seen in Figure A2 that the position where stress begins to develop is where the temperature reaches *T_p_*. When the temperature is lower than *T_p_*, the stress increases primarily because of the rapid increase in material stiffness.

In the fast cooling case, the final residual stresses also exhibit a parabolic shape, and the values at the top and bottom surfaces are −8.75 and 3.96 MPa, respectively, and are approximately twice as large as those of the slow cooling case. The larger value of the residual stress is caused by the faster cooling rate. As the duration of stage 2 is approximately the same in both the fast and slow cooling cases, the faster cooling rate corresponds to a greater difference in the rate of strain, leading to larger residual stress according to Equation (14).

## Appendix B. Plate Thickness

A plate with half thickness cooling at a slow rate is modeled. Figure A3 shows the stress evolution curves at three different positions. The final residual stress at the top and bottom surfaces are −1.17 and 0.59 MPa, respectively. Compared to the thicker plate in Section 5, stage 2 in Figure A3 it has a shorter duration, because the cooling rate is the same and the temperature difference between the top and bottom surfaces is only 2.3 °C. As a result, less residual stress accumulates.
